# Evaluation of a Chronic Pain Screening Program Implemented in Primary Care

**DOI:** 10.1001/jamanetworkopen.2021.18495

**Published:** 2021-07-27

**Authors:** Lauren Bifulco, Daren R. Anderson, Mary L. Blankson, Veena Channamsetty, Jacquelyn W. Blaz, Tam T. Nguyen-Louie, Sarah Hudson Scholle

**Affiliations:** 1 Community Health Center Inc, Middletown, Connecticut; 2 Weitzman Institute, Middletown, Connecticut; 3 Institute for Research and Education to Advance Community Health, Washington State University, Seattle; 4 Aligned Psychotherapy, Columbia, Maryland; 5 National Committee for Quality Assurance, Washington, DC

## Abstract

**Importance:**

Although pain is among the most common symptoms reported by patients, primary care practitioners (PCPs) face substantial challenges identifying and assessing pain.

**Objective:**

To evaluate a 2-step process for chronic pain screening and follow-up in primary care.

**Design, Setting, and Participants:**

A cross-sectional study of patients with a primary care visit between July 2, 2018, and June 1, 2019, was conducted at a statewide, multisite federally qualified health center. Participants included 68 PCPs and 58 medical assistants from 13 sites who implemented the screening process in primary care, and 38 866 patients aged 18 years or older with a primary care visit during that time.

**Exposures:**

Single-question assessment of pain frequency, followed by a 3-question PEG (pain, enjoyment of life, general activity) functional assessment for patients with chronic pain.

**Main Outcomes and Measures:**

Adherence to a 2-step chronic pain screening and PEG process, proportion of patients with positive screening results, mean PEG pain severity greater than or equal to 7, and documented chronic painful condition diagnosis in patient’s electronic health record between 1 year before and 90 days after screening.

**Results:**

Of 38 866 patients with a primary care visit, 31 600 patients (81.3%) underwent screening. Mean (SD) age was 46.2 (15.4) years, and most were aged 35 to 54 years (12 987 [41.1%]), female (18 436 [58.3%]), Hispanic (14 809 [46.9%]), and English-speaking (22 519 [71.3%]), and had Medicaid insurance (18 442 [58.4%]). A total of 10 262 participants (32.5%) screened positive and, of these, 9701 (94.5%) completed the PEG questionnaire. PEG responses indicated severe pain interference with activities of daily living (PEG ≥7) in 5735 (59.1%) participants. A chronic painful condition had not been diagnosed in 4257 (43.9%) patients in the year before screening. A new chronic painful condition was diagnosed at screening or within 90 days in 2250 (52.9%) patients. Care teams found the workflow acceptable, but cited lengthy administration time, challenges with comprehension of the PEG questions, and limited comprehensiveness as implementation barriers.

**Conclusions and Relevance:**

A systematic, 2-step process for chronic pain screening and functional assessment in primary care appeared to identify patients with previously undocumented chronic pain and was feasible to implement. Patient-provided information on the frequency of pain, pain level, and pain interference can help improve the assessment and monitoring of pain in primary care.

## Key Points

**Question:**

What is the utility of using a patient-reported outcome measure to assess chronic pain?

**Findings:**

In this cross-sectional study of 31 600 patients, 32.5% screened positive for chronic pain with use of a tool comprising a single-question assessment of pain frequency, followed by a 3-question functional assessment (pain, enjoyment of life, and general activity). Of these patients, 43.9% had not received a diagnosis for a chronic painful condition and 59.1% reported severe pain interference with activities of daily living.

**Meaning:**

The findings of this study suggest that implementing this measure for chronic pain screening and functional assessment might provide additional clinical information on patients’ pain experience.

## Teaser

This cross-sectional study evaluated a 2-item tool used to assess patients’ pain and its interference with activities of daily living in a primary care setting.

## Introduction

Chronic pain is a leading cause of disability and one of the most common reasons for seeking medical care.^1,2^ In 2016, approximately 50 million US adults (20.4% of the population) experienced chronic pain and 8.0% experienced high-impact chronic pain that limited work or other life activities.^1^ More than a third of US adults with painful health conditions have indicated that pain moderately or severely interferes with activities of daily living.^3^ Women compared with men and African American and Hispanic individuals compared with individuals of other races/ethnicities are more likely to report chronic pain and greater severity of pain,^1^ and prevalence of chronic pain increases with age.^4^

Most people with chronic pain are treated in primary care.^5,6^ Severe chronic pain^7^ disproportionately affects medically underserved patients,^8^ including those with low socioeconomic status, less than a high school educational level, and Medicaid health insurance.^1^ These patients have increased vulnerability to chronic pain^1,9^ and may experience diminished quality of care^10,11^ owing to limited access to treatment,^12,13^ misunderstanding or misinterpretation of pain severity, and misaligned expectations between patients and health care professionals about pain treatment, function, and quality of life.^14,15^ These factors are particularly relevant in Federally Qualified Health Centers (FQHCs), which provide primary care to approximately 28 million medically underserved patients in the US,^16^ including a high proportion of racial and ethnic minority groups.^17^ Pain care delivered at FQHCs is subject to the same quality standards as primary care in other settings,^18^ but FQHC care teams must also contend with limitations in staffing and other resources as well as diminished access to recommended treatment modalities.^19,20^

Although the 11-point Numeric Rating Scale pain score is widely used to assess pain, this approach has limitations^21,22,23^ and improved tools are needed to guide treatment of pain.^24^ The 2016 Centers for Disease Control and Prevention guideline for prescribing opioids for chronic pain^25^ recommends longitudinal pain follow-up using a validated functional assessment tool, such as the PEG,^26^ a patient-reported outcome measure (PROM)^27^ derived from the Brief Pain Inventory,^28,29^ which assesses patients’ self-reported average pain intensity and pain’s interference with enjoyment of life and general activity. PROM tools, such as the PEG, examine health status and health-related quality of life from patients’ perspectives and allow health care professionals to more systematically incorporate patients’ experiences and preferences into their treatment plan.^27,30^ There is limited information on the best approaches for incorporating pain functional assessment in primary care,^31,32^ and available information^26^ may not be generalizable to safety-net practices such as FQHCs. The objective of this study was to evaluate the implementation of a 2-step process in a large, multisite FQHC to screen for chronic pain and assess its association with function as reported by patients.

## Methods

### Study Setting and Participants

This cross-sectional observational study was conducted at a statewide, multisite FQHC in Connecticut providing medical, behavioral health, and dental care to approximately 100 000 patients, most of whom are members of racial/ethnic minority groups, have income less than 200% of the federal poverty level, and reside in urban or suburban areas. Patients were empaneled and cared for by primary care teams consisting of primary care practitioners (PCPs) (family doctors, internists, and nurse practitioners), medical assistants (MAs), nurses, and colocated behavioral health professionals.

The Community Health Center Inc and Advarra institutional review boards reviewed and approved the study protocol. The institutional review boards granted a waiver of consent for analysis of data extracted from medical records and for observation of clinic operations. Professionals provided verbal informed consent for interviews; no financial compensation was provided. This study followed the Strengthening the Reporting of Observational Studies in Epidemiology (STROBE) reporting guideline for observational cross-sectional studies.

Sixty-eight PCPs and 58 MAs from 13 practice sites implemented the new process between July 2, 2018, and June 1, 2019. Patients who participated were aged 18 years or older and presented for a primary care visit during that period. The new process was piloted at a single site for 2 months and then introduced to all practice sites.

### Measurement Tools and Assessment

Clinical decision support tools were developed to align the new process with existing intake and screening workflows. Previously, all patients had been screened at each visit with an 11-point Numeric Rating Scale pain scale. The present study replaced the existing process with a new 2-step process. All PCPs and MAs received live training on the new process in person (pilot site) or via webinar. A custom clinical decision support dashboard prompted MAs to screen all adult patients for chronic pain annually. If a patient had screened positive within the past year and had not completed PEG within the past 90 days, the MA was prompted to administer it. MAs entered patient responses into an electronic data collection smart form in the electronic health record; the responses were stored as part of the patient’s record and were visible to the PCP for discussion during the visit (eFigure in the Supplement).

### Data and Measures

We used a validated, single-question chronic pain screening tool: “In the past 3 months, how often did you have pain?” Answer choices were never, some days, most days, every day, and don’t know. Responses of most days or every day were considered a positive screening indicator for chronic pain.^1,33^ The MAs administered the 3-question PEG^26^ after a positive screen and quarterly (every 90 days) thereafter, until the patient screened negative for chronic pain at subsequent annual chronic pain screening. An average PEG response of greater than or equal to 7 (scale, 0-10) was considered severe pain interference.^28,34,35^ Age, sex, self-reported race/ethnicity, primary insurance, and preferred language were extracted from the electronic health record, corresponding to hypothesized association with potential pain disparities.

The parent study’s project advisory panel of academic pain specialists, frontline clinical professionals, epidemiologists, and health services researchers developed a consensus list of diagnoses associated with painful conditions (eTable in the Supplement). We queried the electronic health record for diagnoses in the year before or in the 90 days after pain screening.

We interviewed the pilot site’s medical director, senior MA, nurse manager, and administrative leaders 6 weeks after launch regarding usability, acceptability, and suggestions for improvement, and incorporated their feedback before launching the new process organizationwide. Two months after organizationwide adoption, 2 observers conducted workflow observations and interviews with care team members at 2 sites to further assess adherence to the planned workflow and obtain additional feedback on the process.

### Statistical Analysis

We conducted bivariate statistical analyses using χ^2^ tests in R, version 3.6.3 (R Foundation). *P* values were adjusted for multiple comparisons using the Holm method; owing to a large sample size, the α level was set to .01 and effect size was measured using the Cramer V statistic. We analyzed qualitative data from key informant interviews using content analysis in NVivo, version 16 (QSR International).

## Results

### Chronic Pain Screening and Functional Assessment

A total of 31 600 patients (81.3% of those with a visit) were screened for chronic pain with the new 2-step process; mean (SD) age was 46.2 (15.4) years and most were aged 35 to 54 years (12 987 [41.1%]), female (18 436 [58.3%]), Hispanic (14 809 [46.9%]), and English-speaking (22 519 [71.3%]), and had Medicaid insurance (18 442 [58.4%]). A total of 13 164 (41.7%) men were included. Of patients assessed, 10 262 (32.5%) screened positive for chronic pain, and 9701 (94.5%) with positive screening results completed PEG functional assessment (Figure).

**Figure.**
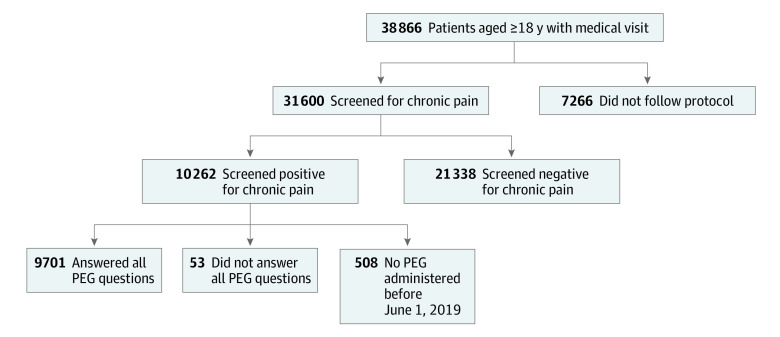
Progression of Patients With Visit Through Intended Workflow Of 38 866 patients with a visit, 31 600 (81.3%) were screened for chronic pain. A total of 10 262 (32.5%) patients screened positive and 9701 (94.5%) of those completed the 3-question functional assessment with the PEG (pain, enjoyment of life, general activity) patient-reported outcome measure.

Most patients who screened positive for chronic pain were aged 35 to 54 years (4674 [45.5%]) (mean [SD], 49.2 [13.7] years), female (6176 [60.2%]), Hispanic (4228 [41.2%]), and English-speaking (8193 [79.8%]), and had Medicaid insurance (6367 [62.0%]). Screening results were moderately associated with age (Cramer V = 0.178; 95% CI, 0.167-0.188; *P* < .001) and weakly associated with race/ethnicity (Cramer V = 0.126; 95% CI, 0.115-0.138; *P* < .001), insurance (Cramer V = 0.134; 95% CI, 0.124-0.145; *P* < .001), preferred language (Cramer V = 0.131; 95% CI, 0.121-0.142; *P* < .001) (Table 1), and the presence of a documented chronic painful condition in the year before chronic pain screening (Cramer V = 0.276; 95% CI, 0.264-0.287; *P* < .001) (Table 2).

**Table 1.** Demographics of Patients Screened for Chronic Pain

Variable	Overall sample (n = 31 600), No. (%)	Screening result, No. %		Cramer V (95% CI)	*P* value^a^
		Negative (n = 21 338)	Positive (n = 10 262)		
Age, y					
18-34	8501 (26.9)	6782 (31.8)	1719 (16.8)	0.178 (0.167-0.188)^b^	<.001
35-54	12 987 (41.1)	8313 (39.0)	4674 (45.5)		
55-64	6253 (19.8)	3545 (16.6)	2708 (26.4)		
≥65	3859 (12.2)	2698 (12.6)	1161 (11.3)		
Sex					
Female	18 436 (58.3)	12 260 (57.5)	6176 (60.2)	0.026 (0.014-0.037)	<.001
Male	13 164 (41.7)	9078 (42.5)	4086 (39.8)		
Race/ethnicity					
Hispanic	14 809 (46.9)	10 581 (49.6)	4228 (41.2)	0.126 (0.115-0.138)^c^	<.001
Non-Hispanic Black	3429 (10.9)	2289 (10.7)	1140 (11.1)		
Non-Hispanic White	8871 (28.1)	5193 (24.3)	3678 (35.8)		
Non-Hispanic other	2641 (8.4)	1968 (9.2)	673 (6.6)		
Other^d^	1850 (5.9)	1307 (6.1)	543 (5.3)		
Primary insurance					
Medicaid	18 442 (58.4)	12 075 (56.6)	6367 (62.0)	0.134 (0.124-0.145)^c^	<.001
Medicare	3254 (11.2)	2039 (9.6)	1485 (14.5)		
Private	5904 (18.7)	4163 (19.5)	1741 (17.0)		
Uninsured	3614 (11.4)	2960 (13.9)	654 (6.4)		
Other public	116 (0.4)	101 (0.5)	15 (0.1)		
Preferred language					
English	22 519 (71.3)	14 326 (67.1)	8193 (79.8)	0.131 (0.121-0.142)^c^	<.001
Spanish	7990 (5.3)	6168 (28.9)	1822 (17.8)		
Other	1091 (3.5)	844 (4.0)	247 (2.4)		

^a^ *P* values adjusted for multiple comparisons using the Holm method; all *P* values significant at <.001 owing to sample size.

^b^ Moderate strength of association.

^c^ Weak strength of association.

^d^ This category included patients who self-reported their race and ethnicity as “American Indian or Alaska Native,” “Asian,” “Multiracial,” “Native Hawaiian or Other Pacific Islander,” “Other,” or who had no race/ethnicity data available.

**Table 2.** Pain Diagnoses of Patients Screened

Variable	Overall sample (n = 31 600), No. (%)	Screening result, No. %		Cramer V (95% CI)	*P* value^a^
		Negative (n = 21 338)	Positive (n = 10 262)		
Documented pain diagnosis in year before screening (n = 31 600)					
Had existing pain diagnosis^b^	11 725 (37.1)	5948 (27.9)	5777 (56.3)	0.276 (0.264-0.287)^c^	<.001
No existing pain diagnosis	19 875 (62.9)	15 390 (72.1)	4485 (43.7)		
Newly documented pain diagnosis among those with no existing pain diagnosis at screening (n = 19 875)					
New pain diagnosis^d^	5232 (26.3)	2862 (18.6)	2370 (52.8)	0.325 (0.310-0.340)^e^	<.001
No new pain diagnosis	14 643 (73.7)	12 528 (81.4)	2115 (47.2)		

^a^ *P* values adjusted for multiple comparisons using the Holm method; all *P* values significant at *P* < .001 owing to sample size.

^b^ Chronic pain condition diagnosis documented within 365 days before screening.

^c^ Weak strength of association.

^d^ No existing pain diagnosis and a documented chronic pain diagnosis on the day of screening or within 90 days postscreening.

^e^ Moderate strength of association.

There were 19 875 patients without a documented chronic painful condition diagnosis in the year before screening. For these patients, their screening result was moderately associated with whether they received a chronic painful condition diagnosis on the date of screening or within 90 days (n = 19 875; Cramer V = 0.325; 95% CI, 0.310-0.340; *P* < .001). Of 19 875 patients without a documented chronic painful condition diagnosis in the year before screening, 4485 (22.6%) screened positive for chronic pain; 2370 (52.8%) of these patients received a new pain diagnosis on the date of their positive screen or within 90 days and 2115 (47.2%) (20.6% of all 10 262 patients who screened positive) did not (Table 2).

Of the 9701 patients who completed PEG (Table 3), 5735 (59.1%) reported severe impairment (PEG ≥7), including more than half of patients in all age groups (eg, 35-54 years: 2653 of 4430 [59.9%]) and with public insurance (Medicare, 867 [61.5%]; Medicaid, 3672 [61.0%]; and other public insurance, 9 [64.3%]), and approximately two-thirds of patients with non-Hispanic Black (710 [65.7%]) or Hispanic (2536 [68.6%]) race/ethnicity or Spanish as the preferred language (1117 [66.7%]). Nearly twice as many patients with severe functional impairment (PEG ≥7) had an existing pain diagnosis (3470 of 5444 [63.7%]) compared with those with less-severe impairment (1974 of 5444 [36.3%]). Those with severe functional impairment without existing diagnoses were also more likely to have a new pain diagnosis documented on the date of screening or within 90 days (1333 of 2250 [59.2%]) (Table 3). All of these factors were weakly associated with severity (*P* < .001 for all).

**Table 3.** Pain Severity Among Patients With Positive Screen Who Completed PEG^a^

Variable	Completed PEG (n = 9701)^b^	PEG result^c^		Cramer V (95% CI)	*P* value^d^
		Not severe (n = 3966)	Severe (n = 5735)		
Age, y					
18-34	1635 (16.9)	782 (19.7)	853 (14.9)	0.065 (0.046-0.086)^e^	<.001
35-54	4430 (45.7)	1777 (44.8)	2653 (46.3)		
55-64	2559 (26.4)	990 (25.0)	1569 (27.4)		
≥65	1077 (11.1)	417 (10.5)	660 (11.5)		
Sex					
Female	5831 (60.1)	2251 (56.8)	3580 (62.4)	0.057 (0.037-0.077)	<.001
Male	3870 (39.9)	1715 (43.2)	2155 (37.6)		
Race/ethnicity					
Hispanic	3697 (40.9)	1431 (36.1)	2536 (44.2)	0.111 (0.092-0.131)^e^	<.001
Non-Hispanic Black	1081 (11.1)	371 (9.4)	710 (12.4)		
Non-Hispanic White	3493 (36.0)	1632 (41.1)	1861 (32.4)		
Non-Hispanic other	639 (6.6)	301 (7.6)	338 (5.9)		
Other^f^	521 (5.4)	231 (5.8)	290 (5.1)		
Primary insurance					
Medicaid	6015 (62.0)	2343 (59.1)	3672 (64.0)	0.076 (0.058-0.098)^e^	<.001
Medicare	1409 (14.5)	542 (13.7)	867 (15.1)		
Private	1650 (17.0)	773 (19.5)	877 (15.3)		
Uninsured	613 (6.3)	303 (7.6)	310 (5.4)		
Other public	14 (0.1)	5 (0.1)	9 (0.2)		
Preferred language					
English	7791 (80.3)	3299 (83.2)	4492 (78.3)	0.071 (0.053-0.092)^e^	<.001
Spanish	1675 (17.3)	558 (14.1)	1117 (19.5)		
Other	235 (2.4)	109 (2.7)	126 (2.2)		
Documented pain diagnosis before screening					
Had existing pain diagnosis^g^	5444 (56.1)	1974 (34.4)	3470 (60.5)	0.106^c^ (0.085-0.125)^e^	<.001
No existing pain diagnosis	4257 (43.9)	1992 (34.7)	2265 (39.5)		
Newly documented pain diagnosis among those without an existing pain diagnosis					
New pain diagnosis^h^	2250 (52.9)	917 (46.0)	1333 (58.9)	0.128 (0.100-0.158)^e^	<.001
No new pain diagnosis	2007 (47.1)	1075 (54.0)	932 (41.1)		

^a^ PEG is a 3-question tool assessing average pain intensity and pain’s interference with enjoyment of life and general activity.

^b^ Included only patients who answered all 3 PEG questions.

^c^ Not severe: level less than 7; severe: level greater than or equal to 7.

^d^ *P* values adjusted for multiple comparisons using the Holm method; all *P* values significant at *P* < .001 owing to sample size.

^e^ Weak strength of association.

^f^ This category included patients who self-reported their race and ethnicity as “American Indian or Alaska Native,” “Asian,” “Multiracial,” “Native Hawaiian or Other Pacific Islander,” “Other,” or who had no race/ethnicity data available.

^g^ Chronic pain condition diagnosis documented within 365 days before screening.

^h^ No existing pain diagnosis and a documented chronic pain diagnosis on the day of screening or within 90 days postscreening.

Workflow observations and interviews identified clinical decision support, time, comprehension, and attitudes about normalizing pain as key themes. Using the clinical decision support dashboard helped facilitate integration of the new process into existing workflows. The MAs initially found it difficult to break the ingrained habit of recording a pain score at every visit in favor of this new 2-step process. PCPs shared concerns about chronic pain screening and PEG results, including “adding a new complaint” that was “usually not part of the [complaint] that the patient is visiting to discuss.”

The PCPs and MAs noted that asking about pain often elicited long responses. An MA stated that “some patients want to give me their life history of pain, and I have to keep reminding them to answer the question”; a PCP commented that, “even when the reason for visit is not pain related, many patients report that they are in pain when asked [and want to discuss it].”

Some patients were unfamiliar with the concept of pain interference and “[did] not understand the PEG questions without examples,” requiring MAs to “break down and simplify” questions to elicit a response. The MAs reported that even after they offered explanations of enjoyment of life and general activity, patients sometimes gave the same numeric response to chronic pain screening and to each PEG question or responded that “[they had] already told [me] their pain was an 8.” One MA speculated that patients may have assumed a higher score would lead to more or better treatment, remarking that “[I have to tell patients that since] we are trying to see whether or not their pain has improved, giving a high score [that does not reflect their actual pain] might mean we stop treatment that’s been working.”

The PCPs believed that the new process increased the need to rediscuss and readdress pain. A PCP noted that “[our] tepid response [to the new process] may be due to pain fatigue—we don’t know what else we can do to treat patients’ pain.” A PCP in a leadership role stated that reframing the discussion about pain would benefit patients and health care professionals, stating that “[though] we have created a culture where we speak about pain a lot, this changes the focus to what really matters: whether pain interferes with activity.”

## Discussion

We used a 2-step screening process to detect chronic pain and assessed its use in a large population of medically underserved patients receiving care in an FQHC. The new process appeared to improve PCPs’ insight into pain interference and pain severity and trigger PCPs to focus on functional assessment.

Approximately one-third (32.5%) of patients in this sample of low-income, urban/suburban, racial and ethnic minority patients screened positive for chronic pain, exceeding the estimated rates of chronic pain in the general population (20%) and in urban areas (16.4%) reported in the 2019 National Health Interview Survey.^7^ In contrast to the National Health Interview Survey, which reported an increasing prevalence of chronic pain with age and higher prevalence of chronic pain among non-Hispanic White adults, we found no significant differences based on race or ethnicity and only a moderate association between chronic pain and patient age. It may be that in this population of vulnerable, medically underserved patients, race, ethnicity, and age interact with socioeconomic factors and other social determinants of health that extend across racial and ethnic groups.^1^ Further studies should examine the extent of this association.

We hypothesized that replacing the use of a 0- to 10-level pain scale at each visit with an annual screening for chronic pain combined with patient self-report of pain frequency and pain interference would improve clinicians’ ability to identify, assess, and monitor chronic pain. Patients’ responses provided clinically useful information that assisted PCPs in detecting and addressing chronic pain. We observed an association between positive vs negative screening results and the presence of an existing pain diagnosis in the year before screening, consistent with earlier studies suggesting that diagnostic codes alone are not useful in differentiating chronic pain from acute or episodic pain.^36^ About half of the patients with a documented pain diagnosis on the date of screening screened positive for chronic pain and half screened negative. A negative screening result may have indicated that pain was well controlled or that the condition was no longer active or causing pain. Future research should examine the medical records of patients who screened negative for chronic pain, despite the presence of a chronic painful condition diagnosis in their medical record within the past year, to assess potential associations with diagnosis and treatment history.

Similarly, 4485 patients (43.7%) with a positive chronic pain screen did not have a previously documented pain diagnosis. The fact that nearly half of them received a new pain diagnosis suggests that the screening process was not simply confirming what was already known to the PCP but rather was flagging issues that may not have been recognized or discussed previously. We were not able to examine whether the 0- to 10-point pain analog scale scores that had been previously recorded were aligned with patients’ self-report of pain most days or every day and their responses to PEG questions about pain intensity and interference with enjoyment of life and general activity. This factor could be a focus of future work.

Augmenting chronic pain screening with PEG helped clinicians identify pain that patients reported as having negative implications in their lives. Nearly 60% of patients who completed PEG reported severe pain interference, far exceeding the National Health Interview Survey estimate of 7.4% of adults in the general population whose pain had frequently limited activities of daily living.^7^ The high prevalence of severe pain detected by this new process confirms the importance of establishing a systematic method for screening and following up on chronic pain.

A significant number of patients who screened positive for chronic pain did not have a documented painful condition diagnosis before screening, nor did they receive one after screening positive (2115 [20.6%]). There are several potential explanations for this lack of diagnosis. Pain, unlike many other chronic conditions, is affected by a range of biopsychosocial factors. Pain may be present without a clear anatomical cause. Our code list for painful conditions did not include psychosocial factors, such as stress, depression, anxiety, or trauma, which are known to affect the pain experience and may result in a report of chronic pain without a pain-specific diagnosis or code. The pain diagnosis guidelines recommended by the International Association for the Study of Pain for the *International Classification of Diseases, Version 11* aim to address this coding gap by including diagnosis codes for classification of chronic pain^37,38^ and providing options to document both chronic primary pain (ie, pain is the principal factor) and chronic secondary pain (ie, pain is a symptom of another condition).^38^

Given that the average primary care visit in the US is between 15 and 20 minutes,^39^ determining what needs to be addressed requires negotiation and balance between what the PCP believes is essential and what the patient wants to discuss. Although adding the chronic pain screen to the routine clinic workflow increased visit complexity, this increase is an inevitable consequence of adding any new screening process in primary care. Clinicians’ frustration and dissatisfaction with having to readdress and rediscuss pain is an understandable consequence of a health care delivery system that is still based largely on short in-person visits and high-volume, fee-for-service encounters. New payment models are needed that reward clinicians both for providing evidence-based care and understanding and supporting person-centeredness. In addition, tools that help patients prepare for their visits in advance and take an active role in setting the agenda may help clinicians better address patients’ most pressing needs within limited visit times.

Using a team-based approach that included MAs was necessary to successful implementation. MAs are typically responsible for data collection and screening and were able to incorporate this new screening process into their existing patient intake process. Familiar clinical decision support tools further facilitated successful implementation.

### Limitations

This study had limitations. We conducted the study at a practice with sophisticated electronic health record data reporting and modification capabilities, which may not be representative of the resources available in all safety-net primary care practices. Other limitations include its focus on a primary care population at a single FQHC, which may not be representative of primary care patients in other settings, and limited ability to assess reasons for nonadherence to the screening protocol or examine patients’ medical records in detail to explain discordance between their screening responses and clinicians’ documentation of a chronic painful condition. Future research should examine patients’ perceptions of this chronic pain screening and functional assessment process to ascertain whether patients feel that pain interference is an important and relevant aspect of their pain and whether they perceive the new process as beneficial to their care.

## Conclusions

This study has implications for primary care. Chronic pain is underdiagnosed and undertreated. We presented a simple, 2-step screening and functional assessment process that might be successfully implemented across a large safety-net practice. The process resulted in the identification of previously undocumented chronic pain conditions and used a PROM tool that helped to engage patients and set the stage for a more patient-centered encounter.

## Notes

Supplement. **eFigure.** Chronic Pain Screening and PEG Workflow Process **eTable.** List of Chronic Pain Diagnoses
