# Supplementary material for: Evaluation of a Chronic Pain Screening Program Implemented in Primary Care
Source: JAMA Netw Open. 2021 Jul 27;4(7):e2118495. doi: 10.1001/jamanetworkopen.2021.18495 (PMC8317006; doi:10.1001/jamanetworkopen.2021.18495)
Supplement: Supplement. — eFigure. Chronic Pain Screening and PEG Workflow Process eTable. List of Chronic Pain Diagnoses [file jamanetwopen-e2118495-s001.pdf]

Supplementary Online Content

Bifulco L, Anderson DR, Blankson ML, et al. Evaluation of a chronic pain screening program implemented in primary care. *JAMA Netw Open*. 2021;4(7):e2118495. doi:10.1001/jamanetworkopen.2021.18495

eFigure. Chronic Pain Screening and PEG Workflow Process

eTable. List of Chronic Pain Diagnoses

This supplementary material has been provided by the authors to give readers additional information about their work.

© 2021 Bifulco L et al. *JAMA Network Open.*

eFigure. Chronic Pain Screening and PEG Workflow Process

Medical assistants administered chronic pain screening and follow-up and documented patient responses in a custom electronic health record smartform for provider and patient to review during the visit.

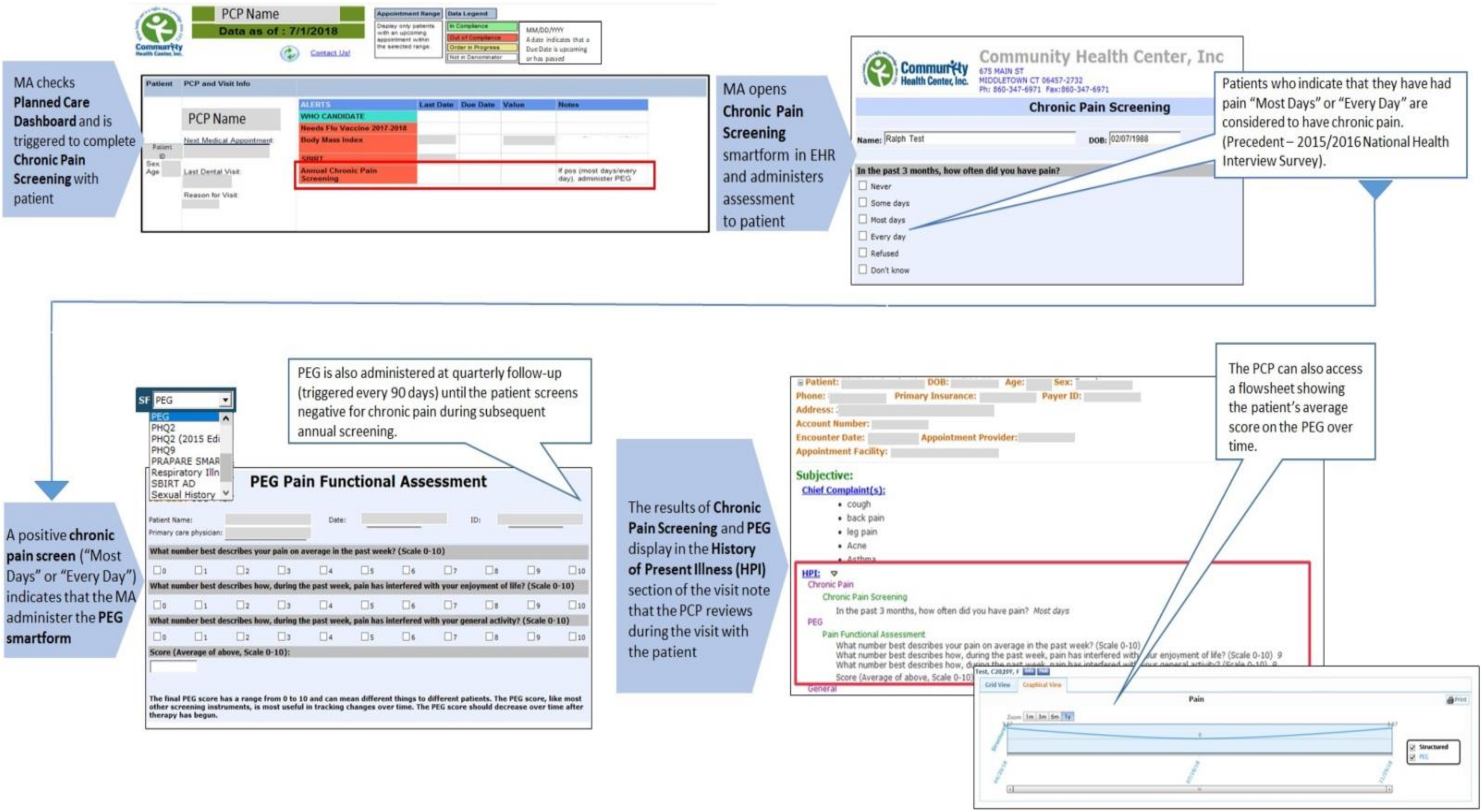

© 2021 Bifulco L et al. *JAMA Network Open.*

eTable: List of chronic pain diagnoses.

ICD	Description
M54.5	Low back pain
M54.41	Lumbago with sciatica, right side
M54.42	Lumbago with sciatica, left side
M51.36	Other intervertebral disc degeneration, lumbar region
M54.16	Radiculopathy, lumbar region
M51.26	Other intervertebral disc displacement, lumbar region
M54.32	Sciatica, left side
M54.31	Sciatica, right side
M54.40	Lumbago with sciatica, unspecified side
M54.30	Sciatica, unspecified side
M51.16	Intervertebral disc disorders with radiculopathy, lumbar region
M53.3	Sacrococcygeal disorders, not elsewhere classified
M47.816	Spondylosis without myelopathy or radiculopathy, lumbar region
M99.03	Segmental and somatic dysfunction of lumbar region
M48.061	Spinal stenosis, lumbar region without neurogenic claudication
M54.17	Radiculopathy, lumbosacral region
M48.06	Spinal stenosis, lumbar region
M96.1	Postlaminectomy syndrome, not elsewhere classified
M99.04	Segmental and somatic dysfunction of sacral region
M47.26	Other spondylosis with radiculopathy, lumbar region
M48.00	Spinal stenosis, site unspecified
M46.90	Unspecified inflammatory spondylopathy, site unspecified
M48.062	Spinal stenosis, lumbar region with neurogenic claudication
M51.37	Other intervertebral disc degeneration, lumbosacral region
M53.86	Other specified dorsopathies, lumbar region
M47.9	Spondylosis, unspecified
M51.27	Other intervertebral disc displacement, lumbosacral region
M46.1	Sacroiliitis, not elsewhere classified

© 2021 Bifulco L et al. *JAMA Network Open.*

ICD	Description
M43.10	Spondylolisthesis, site unspecified
M46.96	Unspecified inflammatory spondylopathy, lumbar region
M47.16	Other spondylosis with myelopathy, lumbar region
M47.896	Other spondylosis, lumbar region
M43.16	Spondylolisthesis, lumbar region
M47.817	Spondylosis without myelopathy or radiculopathy, lumbosacral region
M53.87	Other specified dorsopathies, lumbosacral region
M43.06	Spondylolysis, lumbar region
M48.07	Spinal stenosis, lumbosacral region
M43.17	Spondylolisthesis, lumbosacral region
M47.27	Other spondylosis with radiculopathy, lumbosacral region
M53.2X8	Spinal instabilities, sacral and sacrococcygeal region
M47.897	Other spondylosis, lumbosacral region
M51.06	Intervertebral disc disorders with myelopathy, lumbar region
M43.26	Fusion of spine, lumbar region
M45.5	Ankylosing spondylitis of thoracolumbar region
M46.46	Discitis, unspecified, lumbar region
M46.47	Discitis, unspecified, lumbosacral region
M46.97	Unspecified inflammatory spondylopathy, lumbosacral region
M47.25	Other spondylosis with radiculopathy, thoracolumbar region
M47.818	Spondylosis without myelopathy or radiculopathy, sacral and sacrococcygeal region
M47.895	Other spondylosis, thoracolumbar region
M48.56XA	Collapsed vertebra, not elsewhere classified, lumbar region, initial encounter for fracture
M51.47	Schmorl's nodes, lumbosacral region
M53.2X6	Spinal instabilities, lumbar region
M54.18	Radiculopathy, sacral and sacrococcygeal region
G89.4	Chronic pain syndrome
G89.29	Other chronic pain
Z79.899	Other long term (current) drug therapy

© 2021 Bifulco L et al. *JAMA Network Open.*

ICD	Description
G89.21	Chronic pain due to trauma
Z79.891	Long term (current) use of opiate analgesic
M47.819	Spondylosis without myelopathy or radiculopathy, site unspecified
F45.41	Pain disorder exclusively related to psychological factors
F45.42	Pain disorder with related psychological factors
G89.0	Central pain syndrome
M15.3	Secondary multiple arthritis
R51	Headache
G43.009	Migraine without aura, not intractable, without status migrainosus
G43.909	Migraine, unspecified, not intractable, without status migrainosus
G43.109	Migraine with aura, not intractable, without status migrainosus
G44.209	Tension-type headache, unspecified, not intractable
G43.709	Chronic migraine without aura, not intractable, without status migrainosus
G44.52	New daily persistent headache (NDPH)
G44.89	Other headache syndrome
G43.019	Migraine without aura, intractable, without status migrainosus
G43.119	Migraine with aura, intractable, without status migrainosus
G44.219	Episodic tension-type headache, not intractable
G43.919	Migraine, unspecified, intractable, without status migrainosus
G43.001	Migraine without aura, not intractable, with status migrainosus
G44.229	Chronic tension-type headache, not intractable
G43.101	Migraine with aura, not intractable, with status migrainosus
G44.211	Episodic tension-type headache, intractable
G44.201	Tension-type headache, unspecified, intractable
G44.309	Post-traumatic headache, unspecified, not intractable
G43.809	Other migraine, not intractable, without status migrainosus
G44.019	Episodic cluster headache, not intractable
M71.21	Synovial cyst of popliteal space [Baker], right knee
G44.329	Chronic post-traumatic headache, not intractable
G43.011	Migraine without aura, intractable, with status migrainosus

© 2021 Bifulco L et al. *JAMA Network Open.*

ICD	Description
G43.111	Migraine with aura, intractable, with status migrainosus
G43.509	Persistent migraine aura without cerebral infarction, not intractable, without status migrainosus
G43.901	Migraine, unspecified, not intractable, with status migrainosus
G44.009	Cluster headache syndrome, unspecified, not intractable
G44.221	Chronic tension-type headache, intractable
G44.40	Drug-induced headache, not elsewhere classified, not intractable
G43.519	Persistent migraine aura without cerebral infarction, intractable, without status migrainosus
G44.1	Vascular headache, not elsewhere classified
G44.82	Headache associated with sexual activity
G43.701	Chronic migraine without aura, not intractable, with status migrainosus
G43.819	Other migraine, intractable, without status migrainosus
G44.011	Episodic cluster headache, intractable
G44.039	Episodic paroxysmal hemicrania, not intractable
G44.321	Chronic post-traumatic headache, intractable
G44.59	Other complicated headache syndrome
G43.401	Hemiplegic migraine, not intractable, with status migrainosus
G43.501	Persistent migraine aura without cerebral infarction, not intractable, with status migrainosus
G43.719	Chronic migraine without aura, intractable, without status migrainosus
G43.811	Other migraine, intractable, with status migrainosus
G43.829	Menstrual migraine, not intractable, without status migrainosus
G44.001	Cluster headache syndrome, unspecified, intractable
G44.021	Chronic cluster headache, intractable
G44.029	Chronic cluster headache, not intractable
G44.059	Short lasting unilateral neuralgiform headache with conjunctival injection and tearing
G44.301	Post-traumatic headache, unspecified, intractable
G44.319	Acute post-traumatic headache, not intractable
G44.81	Hypnic headache

© 2021 Bifulco L et al. *JAMA Network Open.*

ICD	Description
G44.85	Primary stabbing headache
M25.561	Pain in right knee
M25.562	Pain in left knee
M25.50	Pain in unspecified joint
M25.511	Pain in right shoulder
M25.512	Pain in left shoulder
M79.671	Pain in right foot
M25.551	Pain in right hip
M79.672	Pain in left foot
M25.552	Pain in left hip
M19.90	Unspecified osteoarthritis, unspecified site
M79.604	Pain in right leg
M17.0	Bilateral primary osteoarthritis of knee
M79.605	Pain in left leg
M79.641	Pain in right hand
M25.569	Pain in unspecified knee
M25.571	Pain in right ankle and joints of right foot
M25.572	Pain in left ankle and joints of left foot
M25.531	Pain in right wrist
M17.11	Unilateral primary osteoarthritis, right knee
M79.642	Pain in left hand
M25.532	Pain in left wrist
M25.521	Pain in right elbow
M77.42	Metatarsalgia, left foot
M77.41	Metatarsalgia, right foot
M79.602	Pain in left arm
M17.12	Unilateral primary osteoarthritis, left knee
M25.519	Pain in unspecified shoulder
M79.601	Pain in right arm
M25.522	Pain in left elbow

© 2021 Bifulco L et al. *JAMA Network Open.*

ICD	Description
M54.10	Radiculopathy, site unspecified
M20.41	Other hammer toe(s) (acquired), right foot
M20.42	Other hammer toe(s) (acquired), left foot
M79.675	Pain in left toe(s)
M79.674	Pain in right toe(s)
M65.4	Radial styloid tenosynovitis [de Quervain]
M16.11	Unilateral primary osteoarthritis, right hip
M77.11	Lateral epicondylitis, right elbow
M75.101	Unspecified rotator cuff tear or rupture of right shoulder, not specified as traumatic
M16.0	Bilateral primary osteoarthritis of hip
M79.644	Pain in right finger(s)
M77.12	Lateral epicondylitis, left elbow
M79.606	Pain in leg, unspecified
M19.011	Primary osteoarthritis, right shoulder
M25.559	Pain in unspecified hip
M23.91	Unspecified internal derangement of right knee
M75.100	Unspecified rotator cuff tear or rupture of unspecified shoulder, not specified as traumatic
M16.12	Unilateral primary osteoarthritis, left hip
M75.102	Unspecified rotator cuff tear or rupture of left shoulder, not specified as traumatic
M79.662	Pain in left lower leg
M79.645	Pain in left finger(s)
M79.673	Pain in unspecified foot
M25.541	Pain in joints of right hand
M65.30	Trigger finger, unspecified finger
M15.9	Polyosteoarthritis, unspecified
M77.10	Lateral epicondylitis, unspecified elbow
M79.661	Pain in right lower leg
M13.0	Polyarthritis, unspecified

© 2021 Bifulco L et al. *JAMA Network Open.*

ICD	Description
M23.92	Unspecified internal derangement of left knee
M67.911	Unspecified disorder of synovium and tendon, right shoulder
M79.609	Pain in unspecified limb
M19.012	Primary osteoarthritis, left shoulder
M25.461	Effusion, right knee
M25.542	Pain in joints of left hand
M70.62	Trochanteric bursitis, left hip
M75.81	Other shoulder lesions, right shoulder
M53.9	Dorsopathy, unspecified
M76.62	Achilles tendinitis, left leg
M79.643	Pain in unspecified hand
M21.611	Bunion of right foot
M25.462	Effusion, left knee
M75.41	Impingement syndrome of right shoulder
M21.612	Bunion of left foot
M70.61	Trochanteric bursitis, right hip
M76.61	Achilles tendinitis, right leg
M12.811	Other specific arthropathies, not elsewhere classified, right shoulder
M15.0	Primary generalized (osteo)arthritis
M65.332	Trigger finger, left middle finger
M67.912	Unspecified disorder of synovium and tendon, left shoulder
M65.311	Trigger thumb, right thumb
M77.32	Calcaneal spur, left foot
M79.622	Pain in left upper arm
M19.041	Primary osteoarthritis, right hand
M21.619	Bunion of unspecified foot
M21.6X1	Other acquired deformities of right foot
M67.431	Ganglion, right wrist
M70.60	Trochanteric bursitis, unspecified hip
M12.9	Arthropathy, unspecified

© 2021 Bifulco L et al. *JAMA Network Open.*

ICD	Description
M25.40	Effusion, unspecified joint
M25.579	Pain in unspecified ankle and joints of unspecified foot
M65.331	Trigger finger, right middle finger
M75.01	Adhesive capsulitis of right shoulder
M20.21	Hallux rigidus, right foot
M23.90	Unspecified internal derangement of unspecified knee
M25.774	Osteophyte, right foot
M75.02	Adhesive capsulitis of left shoulder
M19.049	Primary osteoarthritis, unspecified hand
M21.961	Unspecified acquired deformity of right lower leg
M25.472	Effusion, left ankle
M65.341	Trigger finger, right ring finger
M70.50	Other bursitis of knee, unspecified knee
M71.22	Synovial cyst of popliteal space [Baker], left knee
M75.82	Other shoulder lesions, left shoulder
M79.603	Pain in arm, unspecified
M95.8	Other specified acquired deformities of musculoskeletal system
M12.812	Other specific arthropathies, not elsewhere classified, left shoulder
M19.019	Primary osteoarthritis, unspecified shoulder
M21.6X2	Other acquired deformities of left foot
M22.2X1	Patellofemoral disorders, right knee
M25.539	Pain in unspecified wrist
M25.549	Pain in joints of unspecified hand
M70.21	Olecranon bursitis, right elbow
M77.01	Medial epicondylitis, right elbow
M77.31	Calcaneal spur, right foot
M79.621	Pain in right upper arm
M79.646	Pain in unspecified finger(s)
M79.651	Pain in right thigh
M75.21	Bicipital tendinitis, right shoulder

© 2021 Bifulco L et al. *JAMA Network Open.*

ICD	Description
M75.22	Bicipital tendinitis, left shoulder
M75.42	Impingement syndrome of left shoulder
M19.042	Primary osteoarthritis, left hand
M19.071	Primary osteoarthritis, right ankle and foot
M19.91	Primary osteoarthritis, unspecified site
M20.22	Hallux rigidus, left foot
M21.969	Unspecified acquired deformity of unspecified lower leg
M25.473	Effusion, unspecified ankle
M25.775	Osteophyte, left foot
M25.9	Joint disorder, unspecified
M65.342	Trigger finger, left ring finger
M67.90	Unspecified disorder of synovium and tendon, unspecified site
M15.8	Other polyosteoarthritis
M19.072	Primary osteoarthritis, left ankle and foot
M19.079	Primary osteoarthritis, unspecified ankle and foot
M25.471	Effusion, right ankle
M25.60	Stiffness of unspecified joint, not elsewhere classified
M65.9	Synovitis and tenosynovitis, unspecified
M70.22	Olecranon bursitis, left elbow
M75.112	Incomplete rotator cuff tear or rupture of left shoulder, not specified as traumatic
M21.622	Bunionette of left foot
M21.962	Unspecified acquired deformity of left lower leg
M65.321	Trigger finger, right index finger
M72.0	Palmar fascial fibromatosis [Dupuytren]
M72.9	Fibroblastic disorder, unspecified
M75.122	Complete rotator cuff tear or rupture of left shoulder, not specified as traumatic
M75.51	Bursitis of right shoulder
M77.02	Medial epicondylitis, left elbow
M79.631	Pain in right forearm
M79.652	Pain in left thigh

© 2021 Bifulco L et al. *JAMA Network Open.*

ICD	Description
G90.521	Complex regional pain syndrome I of right lower limb
M12.88	Other specific arthropathies, not elsewhere classified, other specified site
M21.6X9	Other acquired deformities of unspecified foot
M23.204	Derangement of unspecified medial meniscus due to old tear or injury, left knee
M23.8X1	Other internal derangements of right knee
M24.811	Other specific joint derangements of right shoulder, not elsewhere classified
M25.372	Other instability, left ankle
M25.529	Pain in unspecified elbow
M25.619	Stiffness of unspecified shoulder, not elsewhere classified
M65.312	Trigger thumb, left thumb
M67.919	Unspecified disorder of synovium and tendon, unspecified shoulder
M75.121	Complete rotator cuff tear or rupture of right shoulder, not specified as traumatic
M75.52	Bursitis of left shoulder
M76.891	Other specified enthesopathies of right lower limb, excluding foot
M77.30	Calcaneal spur, unspecified foot
M77.52	Other enthesopathy of left foot
M79.669	Pain in unspecified lower leg
G90.522	Complex regional pain syndrome I of left lower limb
M19.032	Primary osteoarthritis, left wrist
M22.2X2	Patellofemoral disorders, left knee
M22.41	Chondromalacia patellae, right knee
M23.8X2	Other internal derangements of left knee
M24.411	Recurrent dislocation, right shoulder
M24.812	Other specific joint derangements of left shoulder, not elsewhere classified
M25.361	Other instability, right knee
M25.362	Other instability, left knee
M25.469	Effusion, unspecified knee
M25.611	Stiffness of right shoulder, not elsewhere classified
M25.641	Stiffness of right hand, not elsewhere classified
M65.319	Trigger thumb, unspecified thumb

© 2021 Bifulco L et al. *JAMA Network Open.*

ICD	Description
M67.449	Ganglion, unspecified hand
M70.52	Other bursitis of knee, left knee
M75.00	Adhesive capsulitis of unspecified shoulder
M75.110	Incomplete rotator cuff tear or rupture of unspecified shoulder, not specified as traumatic
M76.51	Patellar tendinitis, right knee
M76.892	Other specified enthesopathies of left lower limb, excluding foot
M77.00	Medial epicondylitis, unspecified elbow
M77.50	Other enthesopathy of unspecified foot
M79.632	Pain in left forearm
M79.659	Pain in unspecified thigh
G90.511	Complex regional pain syndrome I of right upper limb
G90.523	Complex regional pain syndrome I of lower limb, bilateral
M12.511	Traumatic arthropathy, right shoulder
M12.562	Traumatic arthropathy, left knee
M13.80	Other specified arthritis, unspecified site
M14.679	Charcot's joint, unspecified ankle and foot
M15.4	Erosive (osteo)arthritis
M18.11	Unilateral primary osteoarthritis of first carpometacarpal joint, right hand
M18.12	Unilateral primary osteoarthritis of first carpometacarpal joint, left hand
M19.111	Post-traumatic osteoarthritis, right shoulder
M20.31	Hallux varus (acquired), right foot
M20.32	Hallux varus (acquired), left foot
M20.5X2	Other deformities of toe(s) (acquired), left foot
M21.40	Flat foot [pes planus] (acquired), unspecified foot
M23.206	Derangement of unspecified meniscus due to old tear or injury, right knee
M23.51	Chronic instability of knee, right knee
M24.552	Contracture, left hip
M24.562	Contracture, left knee
M24.571	Contracture, right ankle

© 2021 Bifulco L et al. *JAMA Network Open.*

ICD	Description
M25.30	Other instability, unspecified joint
M25.371	Other instability, right ankle
M25.422	Effusion, left elbow
M25.476	Effusion, unspecified foot
M25.612	Stiffness of left shoulder, not elsewhere classified
M25.642	Stiffness of left hand, not elsewhere classified
M25.649	Stiffness of unspecified hand, not elsewhere classified
M25.662	Stiffness of left knee, not elsewhere classified
M25.80	Other specified joint disorders, unspecified joint
M25.851	Other specified joint disorders, right hip
M65.322	Trigger finger, left index finger
M65.351	Trigger finger, right little finger
M67.432	Ganglion, left wrist
M67.442	Ganglion, left hand
M67.471	Ganglion, right ankle and foot
M67.472	Ganglion, left ankle and foot
M67.98	Unspecified disorder of synovium and tendon, other site
M70.32	Other bursitis of elbow, left elbow
M70.42	Prepatellar bursitis, left knee
M70.51	Other bursitis of knee, right knee
M70.71	Other bursitis of hip, right hip
M71.349	Other bursal cyst, unspecified hand
M75.111	Incomplete rotator cuff tear or rupture of right shoulder, not specified as traumatic
M75.20	Bicipital tendinitis, unspecified shoulder
M75.31	Calcific tendinitis of right shoulder
M75.32	Calcific tendinitis of left shoulder
M75.92	Shoulder lesion, unspecified, left shoulder
M76.01	Gluteal tendinitis, right hip
M76.02	Gluteal tendinitis, left hip

© 2021 Bifulco L et al. *JAMA Network Open.*

ICD	Description
M76.31	Iliotibial band syndrome, right leg
M76.60	Achilles tendinitis, unspecified leg
M76.822	Posterior tibial tendinitis, left leg
M77.40	Metatarsalgia, unspecified foot
M77.51	Other enthesopathy of right foot
M12.529	Traumatic arthropathy, unspecified elbow
M12.551	Traumatic arthropathy, right hip
M12.571	Traumatic arthropathy, right ankle and foot
M12.819	Other specific arthropathies, not elsewhere classified, unspecified shoulder
M12.861	Other specific arthropathies, not elsewhere classified, right knee
M13.161	Monoarthritis, not elsewhere classified, right knee
M14.60	Charcot's joint, unspecified site
M14.671	Charcot's joint, right ankle and foot
M15.2	Bouchard's nodes (with arthropathy)
M18.0	Bilateral primary osteoarthritis of first carpometacarpal joints
M18.10	Unilateral primary osteoarthritis of first carpometacarpal joint, unspecified hand
M18.51	Other unilateral secondary osteoarthritis of first carpometacarpal joint, right hand
M19.021	Primary osteoarthritis, right elbow
M19.022	Primary osteoarthritis, left elbow
M19.031	Primary osteoarthritis, right wrist
M19.131	Post-traumatic osteoarthritis, right wrist
M19.211	Secondary osteoarthritis, right shoulder
M20.001	Unspecified deformity of right finger(s)
M20.002	Unspecified deformity of left finger(s)
M20.011	Mallet finger of right finger(s)
M20.032	Swan-neck deformity of left finger(s)
M20.5X1	Other deformities of toe(s) (acquired), right foot
M21.00	Valgus deformity, not elsewhere classified, unspecified site
M21.069	Valgus deformity, not elsewhere classified, unspecified knee
M21.621	Bunionette of right foot

© 2021 Bifulco L et al. *JAMA Network Open.*

ICD	Description
M21.921	Unspecified acquired deformity of right upper arm
M21.931	Unspecified acquired deformity of right forearm
M21.932	Unspecified acquired deformity of left forearm
M21.941	Unspecified acquired deformity of hand, right hand
M21.949	Unspecified acquired deformity of hand, unspecified hand
M22.42	Chondromalacia patellae, left knee
M23.207	Derangement of unspecified meniscus due to old tear or injury, left knee
M23.209	Derangement of unspecified meniscus due to old tear or injury, unspecified knee
M23.221	Derangement of posterior horn of medial meniscus due to old tear or injury, right knee
M23.251	Derangement of posterior horn of lateral meniscus due to old tear or injury, right knee
M23.307	Other meniscus derangements, unspecified meniscus, left knee
M23.312	Other meniscus derangements, anterior horn of medial meniscus, left knee
M23.321	Other meniscus derangements, posterior horn of medial meniscus, right knee
M23.351	Other meniscus derangements, posterior horn of lateral meniscus, right knee
M23.352	Other meniscus derangements, posterior horn of lateral meniscus, left knee
M23.42	Loose body in knee, left knee
M23.52	Chronic instability of knee, left knee
M23.8X9	Other internal derangements of unspecified knee
M24.20	Disorder of ligament, unspecified site
M24.272	Disorder of ligament, left ankle
M24.311	Pathological dislocation of right shoulder, not elsewhere classified
M24.312	Pathological dislocation of left shoulder, not elsewhere classified
M24.412	Recurrent dislocation, left shoulder
M24.452	Recurrent dislocation, left hip
M24.541	Contracture, right hand
M24.561	Contracture, right knee
M24.572	Contracture, left ankle
M24.573	Contracture, unspecified ankle
M24.80	Other specific joint derangements of unspecified joint, not elsewhere classified

© 2021 Bifulco L et al. *JAMA Network Open.*

ICD	Description
M24.821	Other specific joint derangements of right elbow, not elsewhere classified
M24.849	Other specific joint derangements of unspecified hand, not elsewhere classified
M24.852	Other specific joint derangements of left hip, not elsewhere classified
M24.9	Joint derangement, unspecified
M25.369	Other instability, unspecified knee
M25.411	Effusion, right shoulder
M25.412	Effusion, left shoulder
M25.421	Effusion, right elbow
M25.441	Effusion, right hand
M25.442	Effusion, left hand
M25.449	Effusion, unspecified hand
M25.621	Stiffness of right elbow, not elsewhere classified
M25.661	Stiffness of right knee, not elsewhere classified
M25.739	Osteophyte, unspecified wrist
M25.771	Osteophyte, right ankle
M25.841	Other specified joint disorders, right hand
M25.859	Other specified joint disorders, unspecified hip
M25.861	Other specified joint disorders, right knee
M60.829	Other myositis, unspecified upper arm
M65.20	Calcific tendinitis, unspecified site
M65.28	Calcific tendinitis, other site
M65.359	Trigger finger, unspecified little finger
M65.841	Other synovitis and tenosynovitis, right hand
M67.02	Short Achilles tendon (acquired), left ankle
M67.441	Ganglion, right hand
M67.52	Plica syndrome, left knee
M67.922	Unspecified disorder of synovium and tendon, left upper arm
M70.30	Other bursitis of elbow, unspecified elbow
M70.72	Other bursitis of hip, left hip
M71.20	Synovial cyst of popliteal space [Baker], unspecified knee

© 2021 Bifulco L et al. *JAMA Network Open.*

ICD	Description
M71.312	Other bursal cyst, left shoulder
M71.321	Other bursal cyst, right elbow
M71.332	Other bursal cyst, left wrist
M71.9	Bursopathy, unspecified
M75.40	Impingement syndrome of unspecified shoulder
M76.11	Psoas tendinitis, right hip
M76.12	Psoas tendinitis, left hip
M76.30	Iliotibial band syndrome, unspecified leg
M76.32	Iliotibial band syndrome, left leg
M76.50	Patellar tendinitis, unspecified knee
M76.52	Patellar tendinitis, left knee
M76.71	Peroneal tendinitis, right leg
M76.821	Posterior tibial tendinitis, right leg
M76.829	Posterior tibial tendinitis, unspecified leg
M76.899	Other specified enthesopathies of unspecified lower limb, excluding foot
M79.639	Pain in unspecified forearm
M79.676	Pain in unspecified toe(s)
M79.7	Fibromyalgia
M06.9	Rheumatoid arthritis, unspecified
M10.9	Gout, unspecified
M77.9	Enthesopathy, unspecified
M77.8	Other enthesopathies, not elsewhere classified
M05.79	Rheumatoid arthritis with rheumatoid factor of multiple sites without organ or systems
M45.9	Ankylosing spondylitis of unspecified sites in spine
M05.9	Rheumatoid arthritis with rheumatoid factor, unspecified
M10.072	Idiopathic gout, left ankle and foot
M15.1	Heberden's nodes (with arthropathy)
M06.00	Rheumatoid arthritis without rheumatoid factor, unspecified site
M08.00	Unspecified juvenile rheumatoid arthritis of unspecified site

© 2021 Bifulco L et al. *JAMA Network Open.*

ICD	Description
M10.00	Idiopathic gout, unspecified site
M10.071	Idiopathic gout, right ankle and foot
M10.079	Idiopathic gout, unspecified ankle and foot
M10.09	Idiopathic gout, multiple sites
M11.20	Other chondrocalcinosis, unspecified site
M02.30	Reiter's disease, unspecified site
M05.711	Rheumatoid arthritis with rheumatoid factor of right shoulder without organ or systems involvement
M05.741	Rheumatoid arthritis with rheumatoid factor of right hand without organ or systems involvement
M05.771	Rheumatoid arthritis with rheumatoid factor of right ankle and foot without organ or systems involvement
M05.772	Rheumatoid arthritis with rheumatoid factor of left ankle and foot without organ or systems involvement
M05.89	Other rheumatoid arthritis with rheumatoid factor of multiple sites
M06.021	Rheumatoid arthritis without rheumatoid factor, right elbow
M06.09	Rheumatoid arthritis without rheumatoid factor, multiple sites
M06.30	Rheumatoid nodule, unspecified site
M06.4	Inflammatory polyarthropathy
M10.032	Idiopathic gout, left wrist
M10.069	Idiopathic gout, unspecified knee
M10.472	Other secondary gout, left ankle and foot
M79.2	Neuralgia and neuritis, unspecified
G50.0	Trigeminal neuralgia
G60.9	Hereditary and idiopathic neuropathy, unspecified
M54.81	Occipital neuralgia
G60.3	Idiopathic progressive neuropathy
M54.9	Dorsalgia, unspecified
M54.2	Cervicalgia
M54.6	Pain in thoracic spine
M54.12	Radiculopathy, cervical region

© 2021 Bifulco L et al. *JAMA Network Open.*

ICD	Description
M51.9	Unspecified thoracic, thoracolumbar and lumbosacral intervertebral disc disorder
M50.30	Other cervical disc degeneration, unspecified cervical region
M50.20	Other cervical disc displacement, unspecified cervical region
M50.90	Cervical disc disorder, unspecified, unspecified cervical region
M47.812	Spondylosis without myelopathy or radiculopathy, cervical region
M48.02	Spinal stenosis, cervical region
M47.22	Other spondylosis with radiculopathy, cervical region
M51.34	Other intervertebral disc degeneration, thoracic region
M46.92	Unspecified inflammatory spondylopathy, cervical region
M54.89	Other dorsalgia
M79.12	Myalgia of auxiliary muscles, head and neck
M47.12	Other spondylosis with myelopathy, cervical region
M53.82	Other specified dorsopathies, cervical region
M51.24	Other intervertebral disc displacement, thoracic region
M48.54XA	Collapsed vertebra, not elsewhere classified, thoracic region, initial encounter for fracture
M43.02	Spondylolysis, cervical region
M47.892	Other spondylosis, cervical region
M54.14	Radiculopathy, thoracic region
M25.78	Osteophyte, vertebrae
M43.22	Fusion of spine, cervical region
M43.9	Deforming dorsopathy, unspecified
M46.94	Unspecified inflammatory spondylopathy, thoracic region
M47.815	Spondylosis without myelopathy or radiculopathy, thoracolumbar region
M50.00	Cervical disc disorder with myelopathy, unspecified cervical region
M50.121	Cervical disc disorder at C4-C5 level with radiculopathy
M47.14	Other spondylosis with myelopathy, thoracic region
M47.894	Other spondylosis, thoracic region
M48.03	Spinal stenosis, cervicothoracic region
M48.04	Spinal stenosis, thoracic region

© 2021 Bifulco L et al. *JAMA Network Open.*

ICD	Description
M50.022	Cervical disc disorder at C5-C6 level with myelopathy
M50.10	Cervical disc disorder with radiculopathy, unspecified cervical region
M50.122	Cervical disc disorder at C5-C6 level with radiculopathy
M50.21	Other cervical disc displacement, high cervical region
M50.22	Other cervical disc displacement, mid-cervical region
M50.220	Other cervical disc displacement, mid-cervical region, unspecified level
M50.223	Other cervical disc displacement at C6-C7 level
M50.322	Other cervical disc degeneration at C5-C6 level
M53.80	Other specified dorsopathies, site unspecified
M72.2	Plantar fascial fibromatosis
M79.10	Myalgia, unspecified site
M79.89	Other specified soft tissue disorders
R10.84	Generalized abdominal pain
M79.18	Myalgia, other site
G89.11	Acute pain due to trauma
M79.1	Myalgia
G89.18	Other acute postprocedural pain
M79.9	Soft tissue disorder, unspecified
M1A.9XX0	Chronic gout, unspecified, without tophus (tophi)
M43.6	Torticollis
G89.28	Other chronic postprocedural pain
M1A.0790	Idiopathic chronic gout, unspecified ankle and foot, without tophus (tophi)
M1A.09X0	Idiopathic chronic gout, multiple sites, without tophus (tophi)
M60.9	Myositis, unspecified
M1A.00X0	Idiopathic chronic gout, unspecified site, without tophus (tophi)
M1A.0710	Idiopathic chronic gout, right ankle and foot, without tophus (tophi)
M1A.9XX1	Chronic gout, unspecified, with tophus (tophi)
G50.1	Atypical facial pain
M79.3	Panniculitis, unspecified
N30.10	Interstitial cystitis (chronic) without hematuria

© 2021 Bifulco L et al. *JAMA Network Open.*

ICD	Description
M1A.40X0	Other secondary chronic gout, unspecified site, without tophus (tophi)
G89.12	Acute post-thoracotomy pain
M60.80	Other myositis, unspecified site
G89.22	Chronic post-thoracotomy pain
M1A.00X1	Idiopathic chronic gout, unspecified site, with tophus (tophi)
M1A.0410	Idiopathic chronic gout, right hand, without tophus (tophi)
M1A.0620	Idiopathic chronic gout, left knee, without tophus (tophi)
M1A.0720	Idiopathic chronic gout, left ankle and foot, without tophus (tophi)
M1A.3710	Chronic gout due to renal impairment, right ankle and foot, without tophus (tophi)
M1A.40X1	Other secondary chronic gout, unspecified site, with tophus (tophi)
M1A.4621	Other secondary chronic gout, left knee, with tophus (tophi)
M80.00XD	Age-related osteoporosis with current pathological fracture, unspecified site, subsequent encounter for fracture with routine healing
M80.08XA	Age-related osteoporosis with current pathological fracture, vertebra(e), initial encounter for fracture
M95.0	Acquired deformity of nose
M95.4	Acquired deformity of chest and rib
M95.9	Acquired deformity of musculoskeletal system, unspecified
G89.3	Neoplasm related pain (acute) (chronic)
M42.00	Juvenile osteochondrosis of spine, site unspecified
M43.00	Spondylolysis, site unspecified
M45.0	Ankylosing spondylitis of multiple sites in spine
M46.00	Spinal enthesopathy, site unspecified
M47.10	Other spondylosis with myelopathy, site unspecified
M47.20	Other spondylosis with radiculopathy, site unspecified
M47.899	Other spondylosis, site unspecified
M48.10	Ankylosing hyperostosis [Forestier], site unspecified
M48.9	Spondylopathy, unspecified
M60.89	Other myositis, multiple sites

© 2021 Bifulco L et al. *JAMA Network Open.*

© 2021 Bifulco L et al. *JAMA Network Open.*
